# Apology to the Readers of the Journal of Medicine

**Published:** 1850-08

**Authors:** 


APOLOGY TO THE READERS OF THE JOURNAL OF MEDICINE.

We feel that we owe an apology to the readers of this work, for the delay of the present number, but we feel sure of their forgiveness, when we inform them that imperative duties consequent upon a larger amount of sickness here than usual, accompanied with an epidemic of fright that prompted persons to apply for relief, where there was nothing to relieve, and the heavy and exacting labors imposed upon us, as a member of the Board of Health, are the causes that have postponed the earlier publication of this number. In another part of this Journal, we have given a brief description of cholera in Louisville for 1850, and all well informed medical men will rejoice in the triumphant results of medical hygiene, recorded in those remarks.

